# Bridging therapy with histotripsy prior to liver transplantation for hepatocellular carcinoma: a first case report

**DOI:** 10.1186/s40164-025-00604-z

**Published:** 2025-02-25

**Authors:** Melis Uysal, Chase J Wehrle, Christopher Coppa, Suneel Kamath, Smitha Krishnamurthi, Charles Martin, Mohamed El Hag, Mazhar Khalil, Masato Fujiki, Andrea Schlegel, Charles Miller, Koji Hashimoto, Federico Aucejo, David CH Kwon, Jaekeun Kim

**Affiliations:** 1 Department of General Surgery, Digestive Disease & Surgery Institute, Cleveland Clinic, 9500 Euclid Avenue, Cleveland, OH 44195 USA; 2 Cleveland Clinic, Imaging Institute, Cleveland, OH USA; 3 Department of Oncology, Cleveland Clinic, Taussig Cancer Institute, Cleveland, OH USA; 4 Diagnostics Institute, Department of Pathology, Cleveland Clinic, Cleveland, OH USA; 5 Department of Inflammation and Immunity, Cleveland Clinic, Lerner Research Institute, Cleveland, OH USA

**Keywords:** Histotripsy, Liver transplantation, Hepatocellular carcinoma, Immune response

## Abstract

**Background:**

Histotripsy is a novel, non-invasive, non-ionizing, and non-thermal ablation technique that disrupts tumors using acoustic cavitation. We report the first use of Histotripsy as bridging therapy prior to liver transplant for hepatocellular carcinoma (HCC) treated with histotripsy.

**Case presentation:**

A 59-year-old woman presented with Metabolic-Associated Steatotic Liver Disease (MASLD) cirrhosis (labMELD = 14), hepatic encephalopathy, and a single 2 cm OPTN V lesion in the left lateral segment consistent with HCC. The patient underwent histotripsy treatment of the lesion as bridging therapy before receiving liver transplantation. Histopathology analysis of the explanted liver showed total necrosis of the treated area, with no residual viable tissue tumor.

**Conclusion:**

This case demonstrates the potential utility of histotripsy as an effective bridging therapy for patients with combined cirrhosis and hepatocellular carcinoma (HCC) awaiting liver transplantation, with complete tumor necrosis on explant pathology demonstrating its therapeutic efficacy.

**Supplementary Information:**

The online version contains supplementary material available at 10.1186/s40164-025-00604-z.

To the editor: Liver cancer is a leading cause of cancer-related deaths, with hepatocellular carcinoma (HCC) accounting for over 90% of primary liver cancer cases [1, 2]. The five-year survival rate for liver cancer in the U.S. is only 18%, the second lowest among all cancer types [1]. HCC typically arises in cirrhotic livers and is often diagnosed at advanced stages, although improved screening has led to earlier tumor detection and more locoregional treatment options.

A liver transplant is a potentially curative treatment for hepatocellular carcinoma (HCC), particularly in patients who meet specific criteria, such as the Milan or UCSF criteria [3]. These guidelines ensure the cancer is contained within certain size and number limits, optimizing outcomes post-transplant. Bridging treatments (such as transarterial chemoembolization (TACE), radiofrequency ablation (RFA), or microwave ablation) are often employed to control tumor growth and prevent progression while a patient awaits a transplant [4].

Histotripsy is a novel, non-invasive, non-ionizing, and non-thermal ablation technique that disrupts tumors via controlled acoustic cavitation [5–7]. Worlikar et al. demonstrated its effectiveness in non-invasive liver tumor ablation, showing it prevents local progression and metastasis in rodent models [7]. Histotripsy achieves complete ablation, liquefying target tissue into an acellular homogenate that the body absorbs within two months [8, 9]. The initial THERESA trial reported 100% acute technical success and a 25% incidence of potential off-target, or “abscopal,” effects [10]. The #HOPE4LIVER trial, the largest prospective study on histotripsy to date, included 18 participants, 41% of whom had HCC. Technical success was achieved in 42 out of 44 treated tumors [11].

This case report aims to document the inaugural instance of histotripsy as bridging therapy prior to a liver transplant in a patient with HCC, along with the complete pathologic response of HCC in the explanted liver following histotripsy ablation.

## Case presentation

We report a case of a 59-year-old Caucasian female with morbid obesity (BMI 55 kg/m²) and cirrhosis from metabolic dysfunction-associated steatotic liver disease (MASLD), complicated by hepatic encephalopathy, thrombocytopenia and portal hypertension. An MRI on 8/14/2023 showed a 2 cm LIRAD-5/OPTN-5b lesion consistent with HCC. The patient had an initial AFP of 12.1 ng/ml. The patient underwent evaluation and listed for liver transplantation and the decision was made to pursue bridging therapy first.

While a 6.5% extrahepatic shunt from the hepatic artery to the lungs does not constitute an absolute contraindication to radioembolization, concerns regarding potential pulmonary complications, particularly in the context of the patient’s elevated BMI and planned liver transplantation, warranted careful consideration. Following evaluation by the multidisciplinary tumor board, histotripsy was selected as the optimal therapeutic approach.

**Fig. 1:**
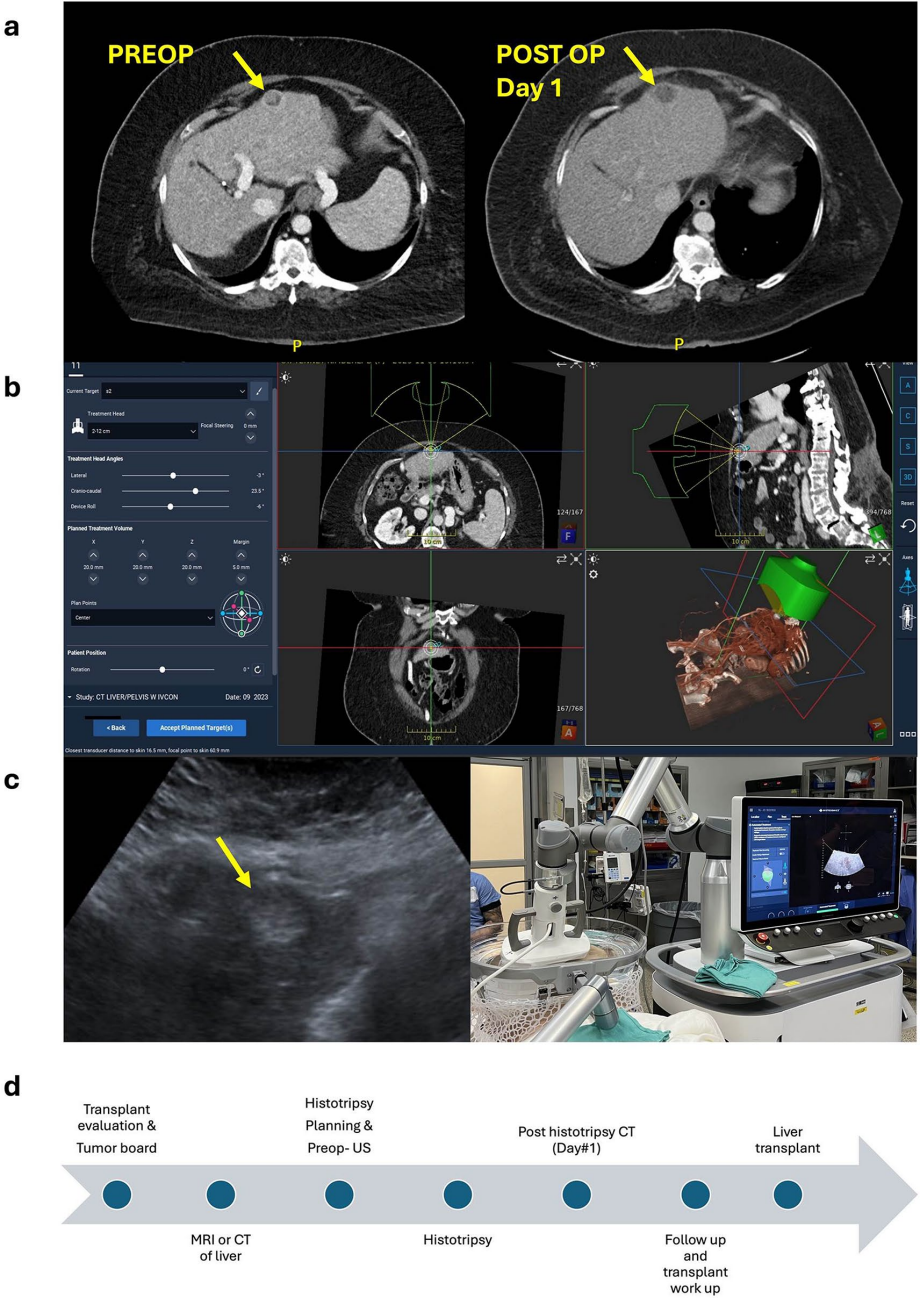
Histotripsy for HCC **a**. Pre & Post histotripsy CT **b**. Histotripsy simulation **c**. Preop US and operation set up **d**. Timeline

The patient underwent liver histotripsy with an ablative margin of 0.5 cm, the patient’s pre- and post-histotripsy CT images can be seen in Fig. 1a. Histotripsy was conducted in an operating room using the HistoSonics Edison System (HistoSonics, Plymouth, MN, USA) under general anesthesia. General anesthesia was necessary to minimize patient movement and ensure controlled respiratory motion during the procedure.

**Fig. 2:**
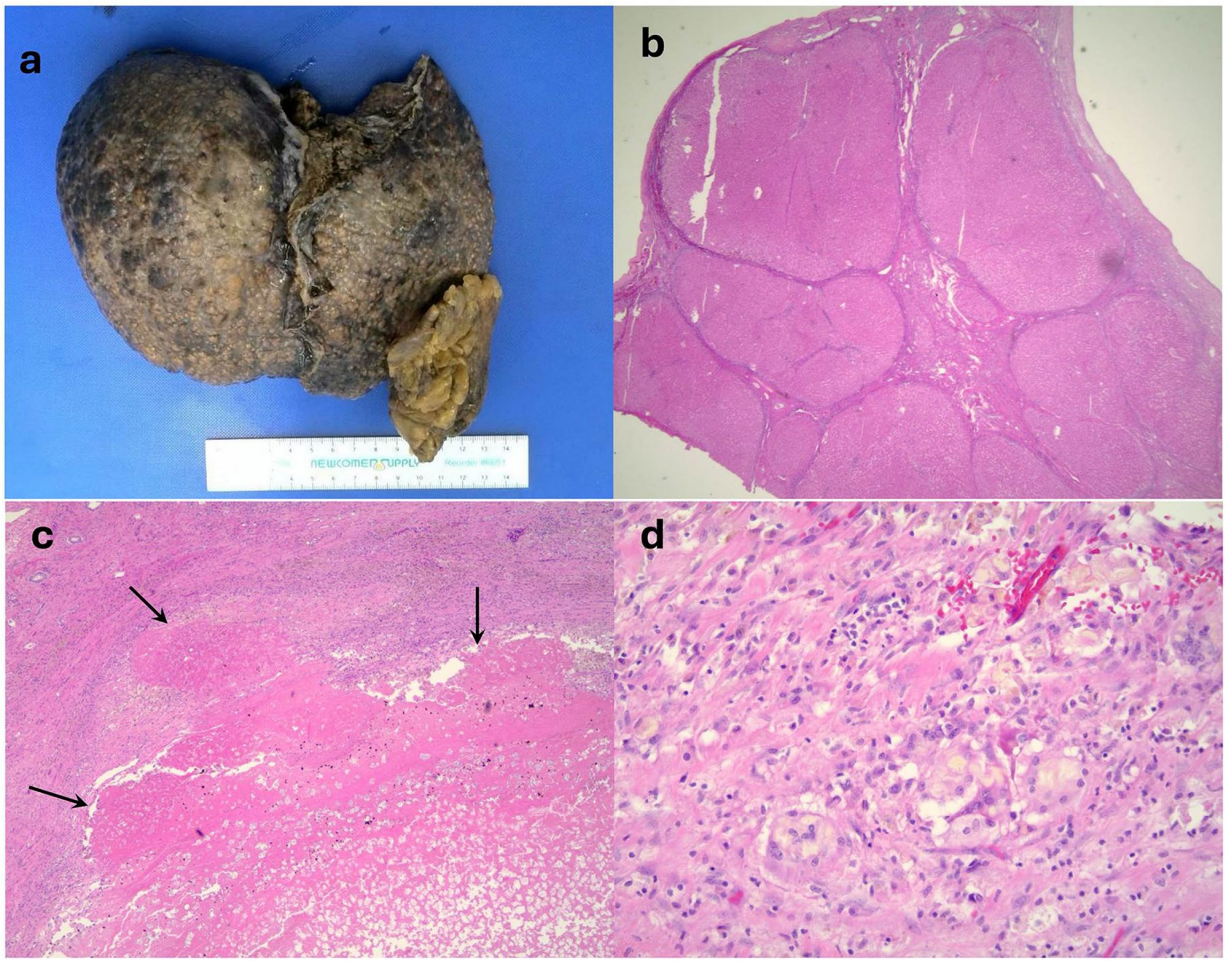
Gross and pathology **a**. explanted liver **b**. untreated cirrhotic nodule (low-magnification, 1.25X) **c**. total necrotic nodule histotripsy treated HCC (below arrows, low-magnification, 4X) **d**. edge of treated HCC showing histiocytes and giant cell reaction (high magnification, 40X)

One month later, she received an orthotopic liver transplant from a donation after cardiac death donor. The explanted liver demonstrated total necrosis of the area treated with histotripsy. The gross image of the macronodular cirrhotic liver can be seen in Fig. 2a. There was no evidence of viable tumor cells at the site of the original tumor on H&E staining and low magnification view of the region of interest showed a necrotic nodule from the treated HCC (Fig. 2c). High magnification of the explanted liver showed histiocytes and giant cell reaction at the edge of the treated tumor (Fig. 2d).

The patient’s postoperative course was complicated by respiratory distress given morbid obesity, fungemia, but she was recovered and discharged to rehab facility postoperative day (POD) 21. Unfortunately, she passed away due to iatrogenic major vascular injury from the rehab facility POD 30.

## Discussion

This report describes the first use of histotripsy as a bridging therapy prior to liver transplantation for HCC, providing post-trial evidence of complete pathological response. While histotripsy is non-invasive and no histological data is available, liver transplantation revealed complete necrosis of the treated lesion. Although human data is lacking, these results align with animal studies by Worlikar et al. [7], which demonstrated complete tumor regression with histotripsy.

Patients with HCC face a significant risk of waitlist drop-out during the six-month waiting period due to tumor progression [4]. Locoregional treatments such as RFA, MWA, TACE, and TARE are well-established options for bridging these patients to liver transplantation [12]. While supported by robust evidence, these modalities have notable limitations, including their invasive nature, significant radiation exposure, limited real-time visualization of therapeutic effects, and inconsistent tumor control. Furthermore, these treatments often necessitate the suspension of systemic therapies, such as chemotherapy or immunotherapy, which can disrupt potential synergistic effects during a critical period. As a result, their use is typically confined to cases with curative intent. In the presented case, conventional bridging methods were not feasible due to hepatic-pulmonary arterial shunting, prompting us to opt for histotripsy ablation. This case highlights the successful use of histotripsy, suggesting it could be a valuable addition to existing HCC treatments, especially given its non-invasive nature and the risks associated with other bridging therapies.

This case report has limitations, including its single-case nature, which may not be representative of broader patient populations. Future research with larger, controlled studies is needed to assess the efficacy of histotripsy in patients with HCC and various liver conditions.

## Conclusion

Histotripsy shows potential as a bridging therapy for HCC. Here, we present the first case of complete tumor necrosis in explanted tissue after histotripsy treatment for HCC, demonstrating its initial use as a bridging therapy before transplantation. Further large-scale studies are needed to validate these findings.

## Electronic supplementary material

Below is the link to the electronic supplementary material.

Supplementary Material 1

## Data Availability

No datasets were generated or analysed during the current study.

## Abbreviations

HCC: Hepatocellular Carcinoma
LT: Liver Transplantation
MWA: Microwave Ablation
IRB: Institutional Review Board
MASLD: Metabolic dysfunction-Associated Steatotic Liver Disease
LIRAD: Liver Imaging Reporting and Data System
OPTN: Organ Procurement and Transplantation Network
POD: Postoperative Day
