# Supplementary material for: Bridging therapy with histotripsy prior to liver transplantation for hepatocellular carcinoma: a first case report
Source: Exp Hematol Oncol. 2025 Feb 25;14:20. doi: 10.1186/s40164-025-00604-z (PMC11863397; doi:10.1186/s40164-025-00604-z)
Supplement: Supplementary file 1 — Supplementary Material 1 [file 40164_2025_604_MOESM1_ESM.docx]

**Figure Legends**

**Figure 1**. **Histotripsy for HCC.** **a** Pre & Postoperative histotripsy CT images. **b** Histotripsy simulation **c** Bubble cloud (6x3x3mm) generated by treatment head of histotripsy and actual operation set up & preoperative US. **d** Histotripsy workflow for transplantation.

**Figure 2.** **Liver transplantation.** **a** Gross image of the explanted liver. H&E-stained sections of the explanted liver: **b** Low-magnification (2X) demonstrating cirrhotic nodules in the untreated liver region.  **c** Low magnification (4X) of the histotripsy-treated tumor, the center of the tumor is necrotic, arrow shows the demarcation between necrotic tumor and background cirrhotic liver.  **d** High-magnification (20X) of the histotripsy-treated tumor showing necrosis, hemorrhage, and macrophages.
